# Dynamics of Bacterial and *Vibrio* Communities in Blacklip Rock Oysters in the Seasonal Tropics

**DOI:** 10.1007/s00248-025-02599-w

**Published:** 2025-11-18

**Authors:** Z. Tinning, M. Kaestli, S. J. Nowland, N. Siboni, J. R. Seymour, K. S. Gibb, A. C. Padovan

**Affiliations:** 1 Research Institute for the Environment and Livelihoods, Charles Darwin University, Darwin, NT Australia; 2 Darwin Aquaculture Centre, Department of Agriculture and Fisheries, Northern Territory Government, Darwin, NT Australia; 3 Climate Change Cluster, University of Technology Sydney, Ultimo, NSW Australia; 4 Australian Centre for Pacific Islands Research, University of the Sunshine Coast, Sippy Downs, Qld Australia

**Keywords:** *Saccostrea spathulata*, Tropical oyster, Host-microbiome, *Vibrio*, Seasonal tropics

## Abstract

Cultivation of the tropical Blacklip Rock Oyster (BRO) (*Saccostrea spathulata*) is an emerging Indigenous-led aquaculture industry in the seasonal tropics of northern Australia. However, little is currently known about the potential for pathogen outbreaks in this species. We conducted a year-long study to establish a microbial baseline to identify potential oyster and human health risks to inform future food safety decision making in this nascent industry. In healthy oysters, we identified both the core microbiome of this oyster species and the presence of potential oyster and human pathogens. The core bacteriome comprised nine bacterial families, while the core vibriome comprised the animal pathogens *Vibrio harveyi* and *V. owensii*. The potential human pathogen *V. parahaemolyticus* was detected in some oysters during the wet season, during periods of increased rainfall, turbidity and total nitrogen. The bacteriome and vibriome of oysters were significantly different to the adjacent seawater and therefore we concluded that seawater is not an appropriate surrogate for pathogen risk surveillance in this developing industry. These results provide new knowledge on the microbiology of a previously understudied oyster species and will inform monitoring methods, harvesting and shellfish quality compliance in this emerging Indigenous-led industry.

**Supplementary Information:**

The online version contains supplementary material available at 10.1007/s00248-025-02599-w.

## Introduction

Globally, the oyster aquaculture industry is facing substantial food safety and animal health concerns, which are often linked to increasing impacts of pathogenic bacteria and environmental change [1–3]. Environmental shifts, such as changes in water temperature, pH and salinity, can provide ideal conditions for opportunistic pathogens to thrive whilst simultaneously causing the animal stress [2]. Due to the filter-feeding nature of oysters, they can assimilate potential oyster and human pathogens thereby disrupting the oyster bacteriome which, during periods of environmental stress, can also coincide with suppressed oyster immune system, leading to detrimental effects on both oyster and human health [4]. The oyster microbiome is increasingly recognised as functionally important to host health, development, and environmental interactions. Core microbiota in oysters are often conserved across individuals and environments, suggesting both ecological [5–7] and physiological significances [8, 9]. Some of the most notable oyster pathogens are members of the *Vibrio* genus, which are associated with warm seawater temperatures and low salinity [10]. Worldwide reports of vibriosis mortalities in oysters are most notably caused by *V. harveyi*, *V. splendidus*, *V. coralliilyticus* and *V. alginolyticus* [11–14].

In Australia, there have also been several reported outbreaks of illness in humans due to the consumption of raw oysters, with most cases caused by *V. parahaemolyticus* [15]. *Vibrio parahaemolyticus* abundance has been shown to exhibit seasonal variations in its abundance in seawater [16] and has been associated with high salinity and chlorophyll *a* [17]. *Vibrio parahaemolyticus* has most recently been linked to a nation-wide outbreak of vibriosis which was traced back to the consumption of Pacific Oysters farmed in South Australia [18]. Climate change is expected to intensify oyster mortalities and increase human infections from oyster consumption, posing a significant economic challenge to the oyster industry [19].

The tropical Blacklip Rock Oyster (BRO), *Saccostrea spathulata* [20, 21], is native to northern Australia and southeast Asia. A nascent BRO industry in the monsoonal tropics of northern Australia has significant potential for the economic development of Indigenous communities that are leading this emerging aquaculture enterprise [22]. However, marine environments in northern Australia are characterised by abundant and diverse *Vibrio* communities [23]. While there have not yet been any reports of oyster mortalities or human disease outbreaks from BROs, farmed and wild BROs in northern Australia host a diverse *Vibrio* community, including the potentially pathogenic species *V. parahaemolyticus* and *V. harveyi* [24]. We posit that the presence of these species in BROs could have implications for shellfish quality assurance and compliance.

This study aims to characterise the seasonal dynamics of the BRO microbiome and in doing so identify the core bacteriome and vibriome of the tropical BRO, as well as identifying putative oyster and human pathogens. We hypothesise that BROs will possess a core microbiome, as well as a diverse transient bacteriome and vibriome, that will change with season. The ‘microbial baseline’ defined by this work has the potential to underpin future decision-making about oyster health, oyster harvest, human health risks and shellfish quality assurance programs in this growing indigenous-led aquaculture industry.

## Methods

### Sample Collection and Processing

Samples were collected by boat during high tide from a remote Blacklip Rock Oyster (BRO) farm located on South Goulburn Island (S11.622071 E133.403951) in the Northern Territory, Australia, at six time points from September 2021 to September 2022 (Sept 2021, Dec 2021, Jan 2022, Apr 2022, Aug 2022, Sept 2022). Blacklip Rock Oysters (mean shell length = 73.3 ± 7.7 mm; mean shell width = 50.2 ± 5.3 mm) and seawater were collected in triplicate from three sites within the farm (approximate distances between sites A, B and C: A to C = 30 m, C to B = 14 m, and B to A = 23 m), representing three different positions across the intertidal longline that is 20 m from the shoreline (*n* = 27 oysters per sampling round with 3 oysters pooled for one sample). Seawater (1 L) was collected from the surface with sterile bottles and oysters were placed in plastic ziplock bags, before being transported to the laboratory at ambient temperatures and processed within 4 hours. A Multi-Parameter Testr 35 Series (Eutech Instruments, Singapore) was used by the oyster growers to measure seawater temperature, pH and salinity on-site at the time of sampling. Salinity data was subsequently found to be unreliable so were excluded from the study. Seawater turbidity was measured using a HYDROLAB® Quanta® (Hydrolab Corporation®, USA) water quality instrument.

Seawater samples (50 mL) were analysed for total nutrients. Total phosphorus (TP) (reporting limit 0.003 mg/L) and total nitrogen (TN) (reporting limit 0.02 mg/L) were measured by persulphate digestion (Forensic and Health Services, Qld Govt). Chlorophyll *a* was measured by fluorometric detection adapted from the Trilogy® Laboratory Fluorometer (Turner Designs, USA) and standard acetone extraction methods (APHA, 2005), following filtration of 500 mL onto glass fibre filters. Stock concentrations of chlorophyll *a* (Sigma-Aldrich, USA) were used for calibration. The Trilogy® Laboratory Fluorometer has an extracted chlorophyll *a* minimum detection limit of 0.0225 μg/L and a reporting limit of 0.1 μg/L.

For seawater microbiome analysis, 500 mL seawater samples were filtered through 0.2 μm mixed cellulose ester filters (Advantec®, Taiwan), which were then stored at -80 °C. Oysters were scrubbed with potable water and shucked using an autoclaved shucking knife. The meat and liquor from three oysters were pooled to give one sample, creating nine biological samples per time point. Each oyster sample was homogenized using an Ultra-Turrax® IKA T18 (IKA® Works, Malaysia) and stored at -80 °C. The average condition of each batch of oysters was assessed visually at each time point by a BRO industry expert, using a qualitative scale ranging from 0 to 100%. This scale reflects condition based on body fat cover, mantle appearance, and shell fullness. A score above 70% indicates oysters are in excellent marketable condition, with full, well-coloured mantles and tissue that fills the shell. Scores below 50% reflect oysters not fit for sale, typically showing reduced body fat and recessed mantles.

Seawater DNA was extracted from filters using the FastDNA™ SPIN Kit for Soil (116,560,200-CF, MP Biomedicals, USA) following the manufacturer’s instructions. For oyster samples, DNA was extracted from approximately 25 mg oyster tissue homogenate using the DNeasy® Blood and Tissue kit (69,504, Qiagen, Germany). Nucleic acid quantity and quality was determined spectrophotometrically with a NanoDrop™ One (Thermo Fisher Scientific, USA) for all samples.

*Vibrio* abundance was quantified using quantitative PCR (qPCR) with the *Vibrio*-specific 16S rRNA gene primers Vib1-f (5’- GGCGTAAAGCGCATGCAGGT -3’) and Vib2-r (5’- GAAATTCTACCCCCCTCTACAG -3’) [25, 26]. Standard curves with seven dilution points and three replicates per dilution were prepared using a known concentration of DNA extracted from a *V. parahaemolyticus* culture (ATCC® 17,802™, 5.15 × 10⁶ bp, Thermo Fisher Scientific, USA) and measured by a Nanodrop™ spectrophotometer prior to diluting. The reaction mixture for each assay included: 10 µL PerfeCTa SYBR® Green SuperMix (Quantabio, USA), 0.8 µL of each 4 mM forward and 4 mM reverse primer, 2 µL of template DNA, and 6.4 µL of sterile water for a final reaction volume of 20 µL. *Vibrio*-specific 16S rRNA gene qPCR was performed using the following cycling conditions: 95 °C for 3 min followed by 40 cycles of 95 °C for 15 s and 60 °C for 1 min, followed by a melting curve to confirm the amplification of only a single product. qPCR data were normalised to tissue weight for oyster samples and volume filtered for seawater.

### Bacterial 16S rRNA Gene and Vibrio-Centric hsp60 Amplicon Sequencing and Analysis

The bacterial community in oyster and seawater samples was determined by 16S rRNA gene amplicon sequencing. Extracted DNA from oyster and seawater samples were amplified by PCR using the Bakt_341F and Bakt_805R primers targeting the V3-V4 region of the prokaryotic 16S rRNA gene [27]. The *Vibrio* community within oysters and seawater was characterised using a *Vibrio*-centric amplicon sequencing assay employing the *hsp60* primers Vib-hspF3-23 and Vib-hspR401-422 [28]. Amplicons were sequenced using the Illumina NovaSeq SP 500 platform according to the manufacturer’s guidelines (Ramaciotti Centre for Genomics, Australia). Raw data files in FASTQ format have been deposited in NCBI Sequence Read Archive (PRJNA1264689).

The 16S rRNA gene paired-end sequences were demultiplexed using QIIME2 (2022.8) (https://qiime2.org/) and the DADA2 plugin within QIIME2 was used to denoise the sequences and assign the amplicon sequence variants (ASVs). During this process, sequences were truncated at 257 bp for both forward and reverse reads to remove primers, and chimeric sequences were also filtered out. The classify-sklearn feature in QIIME2 was used to assign taxonomy (Silva v128 database) to sequences. ASVs that were identified as chloroplasts, mitochondria and unassigned bacteria were removed in QIIME2. ASVs that contributed to < 0.001% of reads were removed, and clean data was rarefied to 18,000 reads per sample, which excluded two oyster samples due to insufficient data (December 2021, *n* = 2).

The quality of *hsp60* amplicon sequences was assessed using FastQC (https://www.bioinformatics.babraham.ac.uk/projects/fastqc/) and due to the low quality of the reverse sequences, only the forward sequences were processed using QIIME2. DADA2 was used to denoise the sequences (left trim 4 bp; length truncation 251 bp; max. expected error 2) and create ASVs. The taxonomy was assigned to the sequence variants in a two-step process based on the *Vibrio hsp60* database (repset_final_130219) from King et al. [28]. The first step used the Blast taxonomy classifier (https://blast.ncbi.nlm.nih.gov/Blast.cgi) set to 90% identity to filter for *Vibrio* sequences and exclude all non-*Vibrio* sequences, while the second step used the sklearn-based taxonomy classifier to identify *Vibrio* species. PhyloSeq [29] in R (v4.2.2; R Core Team 2023) was used to exclude *hsp60* ASVs with a maximum relative abundance < 0.1% or samples which had < 100 reads. This excluded two seawater (December 2021, *n* = 1; January 2022, *n* = 1) and seven oyster samples (September 2021, *n* = 1; December 2021, *n* = 4; January 2022, *n* = 1) due to insufficient data, resulting in ninety-nine samples with reads ranging from 110 to 350,407. Rarefaction curves were conducted which indicated that the ASV richness was reached for all samples with a distinct flattening of curves and the relative ASV abundance was used for ordination and PERMANOVA analyses.

### Statistical Analysis

Bacterial 16S rRNA gene and *Vibrio*-centric *hsp60* gene sequences were analysed in R [29] using the PhyloSeq package (v4.2.2; R Core Team 2023) and Primer-e v7 (Quest Research Limited, Plymouth UK). Differences in environmental variables across months and seasons were assessed using the Kruskal–Wallis rank-sum test performed in R. To assess differences in the bacterial and *Vibrio* communities over time and between seawater and oyster samples, the relative abundance of ASVs was calculated and the Bray Curtis dissimilarity matrix generated. The matrix was visualized with nMDS ordinations. A PERMANOVA and a PermDisp were conducted in Primer-e with the fixed factors of sampling period (*n* = 6 levels), sample type (*n* = 2 – seawater versus oyster) and site (*n* = 3). All main and pairwise comparisons involved 990 + unique permutations. To assess which bacterial families and *Vibrio* species mainly contributed to changes over time and sample type, a “Similarity Percentages—species contributions” (SIMPER) analysis was conducted in Primer-e.

To assess correlations between the seawater and oyster bacterial families and *Vibrio* species community and measured environmental variables, redundancy analysis (RDA) was performed using the package vegan in R. This analysis was at family level for bacteria and species level for *Vibrio*. Rare families/species which occurred in less than three samples were excluded. The RDA analyses used Hellinger transformed (square root of relative abundance) data. The abiotic seawater variables were assessed for skewness and collinearity using histograms, scatter plot matrices and the variance inflation factor.

To identify core ASVs shared between oyster sequence data were analysed using the PhyloSeq package in R. There is currently no consensus on the appropriate prevalence threshold for defining the core microbiome, with recent studies using values ranging from 50 to 85% [6, 7, 9, 30]. In this study, a core ASV was defined as one that occurred in at least 80% of all oyster samples, with a sample consisting of three pooled oysters. The threshold was selected to identify taxa that are highly conserved and stable across samples, while allowing for natural variation across sites and time points, in line with the approach used by King et al. [6].

## Results

### Seasonally Driven Changes in Seawater Chemistry

Seawater temperatures ranged from 27–32 °C, with the coolest period in August, during the dry season, and the warmest period in December, at the beginning of the wet season (Table 1). However, seawater temperature during the January 2022 sampling event was notable for being unseasonably cool, which was likely due to rainfall on the sampling day (Supplementary Fig. 1). Monthly rainfall differed between months, ranging from 27.6 to 332.6 mm during the wet season and from 0 to 4.6 mm during the dry season. There was little variation in pH during the sampling periods with 0.2 pH unit difference between the highest and lowest values, with the highest in the dry season months. Turbidity ranged between 2.7 and 9.5 NTU, with the highest in December 2021 (Table 1). Chlorophyll *a* remained relatively constant for all sampling periods, except for low levels in August 2022. Total phosphorus (TP) values ranged from 0.006 mg/L to 0.022 mg/L and TN values ranged from 0.09 mg/L to 0.16 mg/L, with the highest TP and TN values recorded in December 2021 (Table 1). Kruskal–Wallis tests showed significant differences in environmental parameters by both seasons and between time points. By season, temperature (*p* = 0.015), pH, turbidity, TN and TP (*p* < 0.001) differed significantly between dry and wet seasons, whereas chlorophyll *a* did not (*p* = 0.815). All environmental parameters displayed significant monthly variation (*p* < 0.001), including chlorophyll *a* (*p* < 0.001).

**Table 1:** Average ± standard deviation (SD) physicochemical variables measured in seawater during 6 sampling events at Goulburn Island

Date	Seawater temperature (°C)	pH	Turbidity (NTU)	Chlorophyll *a* (μg/L)	TP (mg/L)	TN (mg/L)	Cumulative rainfall (mm, 30 days before sampling)
13 Sept 2021	30.0 ± 0.0	8.3 ± 0.0	4.1 ± 0.1	1.27 ± 0.10	0.008 ± 0.002	0.15 ± 0.02	0
2 Dec 2021	31.8 ± 0.2	8.1 ± 0.0	9.5 ± 0.0	1.14 ± 0.86	0.022 ± 0.005	0.16 ± 0.03	27.6
27 Jan 2022	29.7 ± 0.1	8.1 ± 0.0	5.8 ± 0.0	1.26 ± 0.39	0.012 ± 0.002	0.13 ± 0.01	147.6
27 Apr 2022	30.7 ± 0.1	8.1 ± 0.0	5.5 ± 0.5	0.98 ± 0.14	0.014 ± 0.002	0.14 ± 0.02	97.8
30 Aug 2022	27.5 ± 0.1	8.2 ± 0.0	4.6 ± 0.2	0.37 ± 0.01	0.008 ± 0.001	0.11 ± 0.01	0
27 Sept 2022	30.8 ± 0.2	8.3 ± 0.0	2.7 ± 1.1	1.35 ± 0.14	0.006 ± 0.001	0.09 ± 0.03	0

### Oyster Condition

The average oyster condition of each batch changed between sampling periods. Oyster batches were in optimum condition in the dry season months August and September (90–100%), but in poor condition in the wet season months of January (10%), April (30%) and December (60%).

### The Oyster Bacteriome and Vibriome Were Different to That in Seawater

**Fig. 1:**
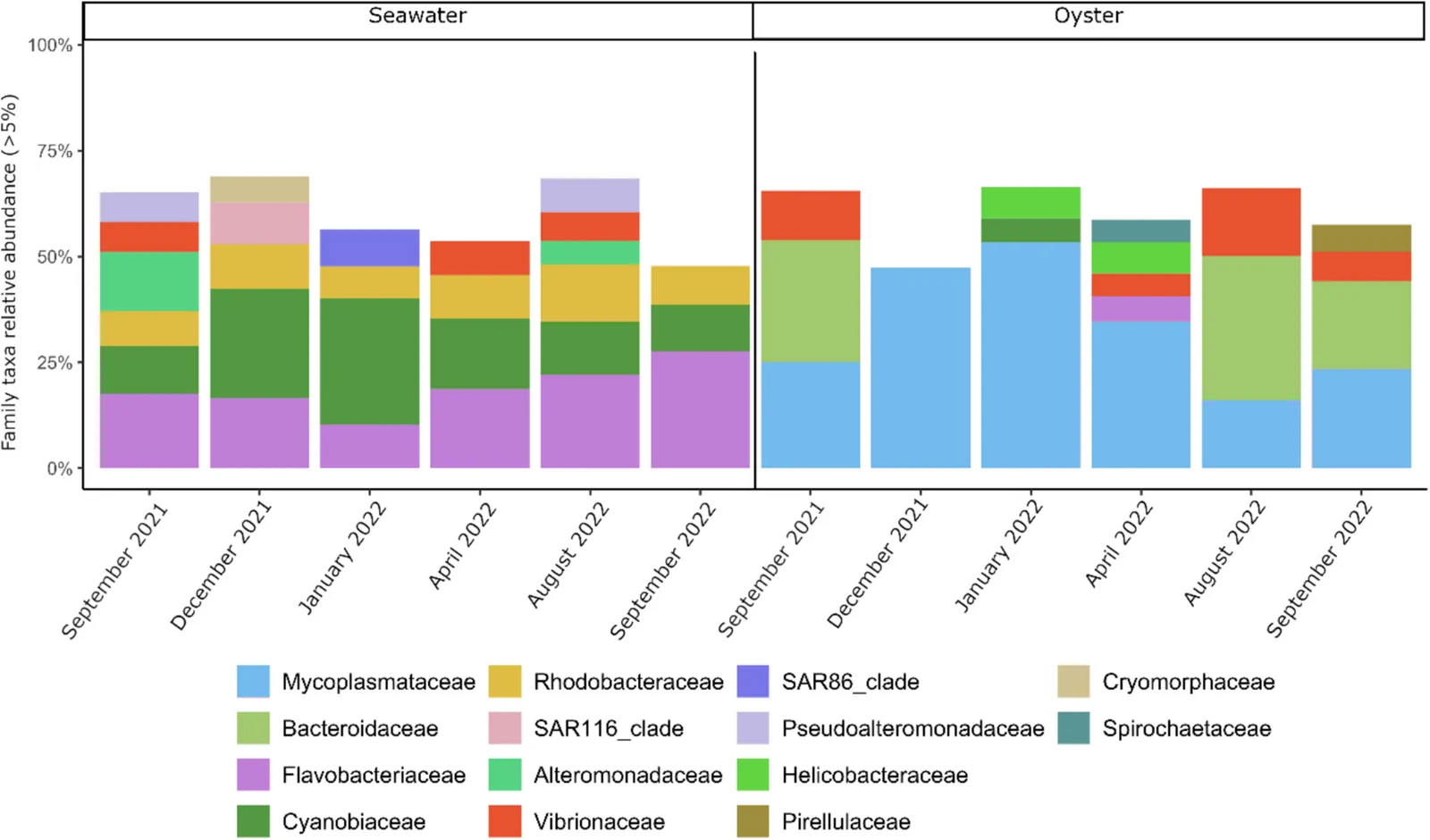
Dominant (> 5%) bacterial species in oyster and seawater at each time point characterised. Following initial sequence data processing, there were 106 samples with 31,183 bacterial 16S rRNA gene ASVs. Bar plots represent the relative abundance of bacterial taxa averaged across the nine pooled oyster samples per time point. Some samples were excluded due to insufficient data. Otherwise, all sample types (*n* = 9) were represented at each time point. Excluded samples were December 2021 (*n* = 2)

The most relatively abundant bacterial families in seawater and oysters differed (Fig. 1). The dominant families in oysters were *Mycoplasmataceae* (30%), *Bacteroidaceae* (14%) and *Vibrionaceae* (7%), while the dominant families in seawater were *Flavobacteriaceae* (18%)*, Cyanobiaceae* (18%) and *Rhodobacteraceae* (9%)*.*

**Fig. 2:**
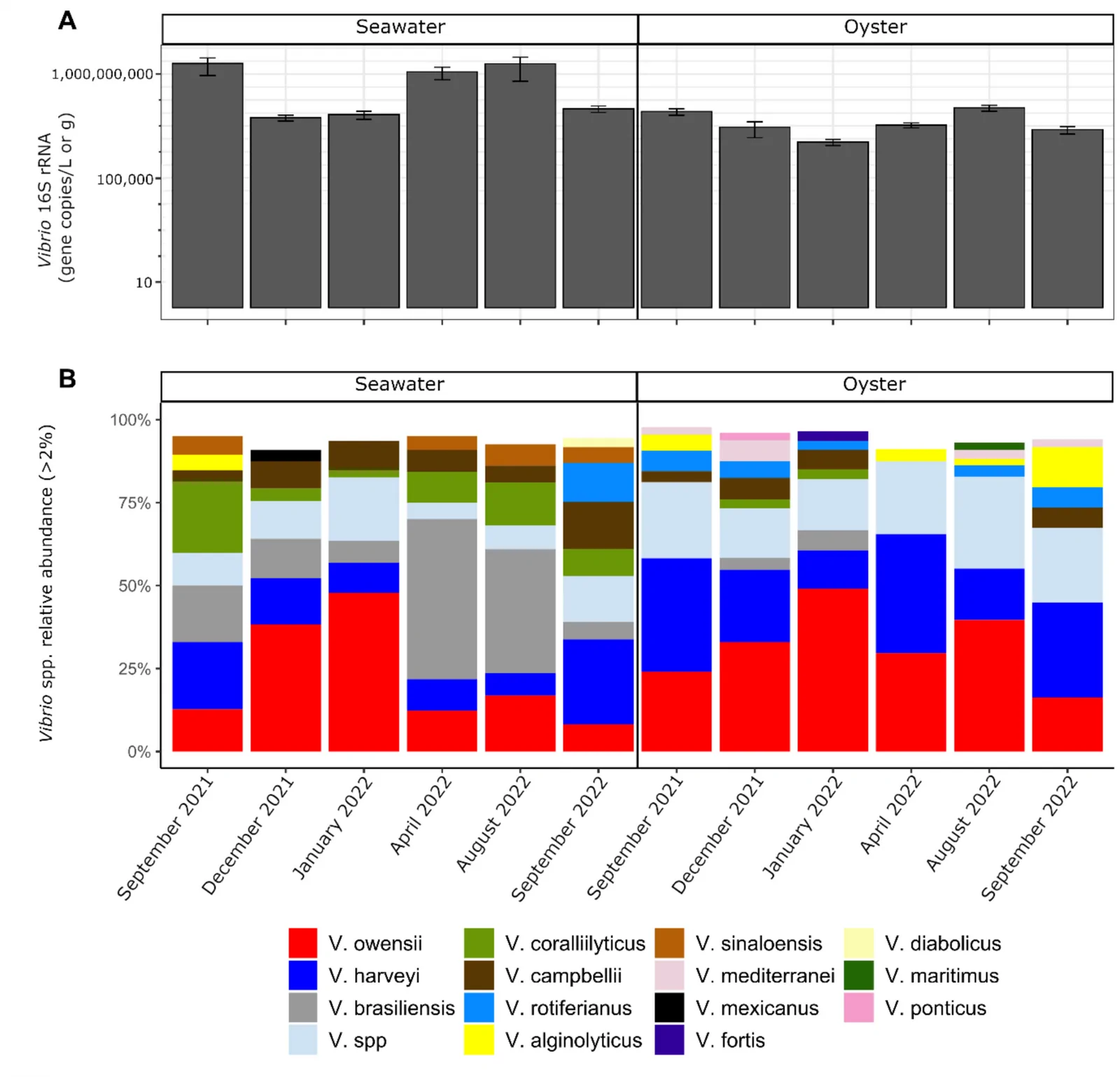
(**A**) *Vibrio* abundance by qPCR measured in copies per litre for seawater and copies per gram for oysters at each time point. Y-axis is in log-10 scale. (**B**) Dominant (> 2%) *Vibrio* species in oyster and seawater samples at each time point. Following initial sequence data processing, there were 99 samples with 763 *Vibrio* hsp60 ASVs, representing 35 *Vibrio* species. Bar plots represent the relative abundance of *Vibrio* species averaged across the nine pooled oyster samples per time point. Some samples were excluded due to insufficient data. Otherwise, all sample types (*n* = 9) were represented at each time point. Excluded samples included December 2021 (*n* = 1) and January 2022 (*n* = 1) seawater samples, and September 2021 (*n* = 1), December 2021 (*n* = 4) and January 2022 (*n* = 1) oyster samples

*Vibrionaceae* relative abundance was lower in both seawater and oysters in December 2021 and January 2022, which was congruous with *Vibrio* 16S rRNA qPCR abundance (Fig. 2A). Oysters and seawater contained 35 *Vibrio* species (Supplementary Table 1), and the dominant (> 5% relative abundance) species were *V. owensii*, *V. harveyi*, *V. brasiliensis*, *V. coralliilyticus* and *V. campbellii*. *Vibrio owensii* was abundant in both seawater and oysters, particularly in the wet season samples of December 2021 and January 2022**.** *Vibrio harveyi* was regularly detected in both seawater and oysters, but its relative abundance was higher in oysters, particularly in the late dry season sample September 2021 and the wet season sample April 2022. *Vibrio brasiliensis*, *V.* *coralliilyticus* and *V. campbellii* mainly occurred in seawater, with *V. brasiliensis* dominating seawater in the wet season sample April 2022 and the dry season sample August 2022 (Fig. 2B). While not abundant, *V. alginolyticus* occurred in seawater and oysters in September 2021 and September 2022, and also in oysters in April 2022 and August 2022. *Vibrio fortis* was detected in oysters in January 2022 (up to 20%) and in seawater in August and September (up to 10%), but was not detected or < 3% at other sample times.

**Fig. 3:**
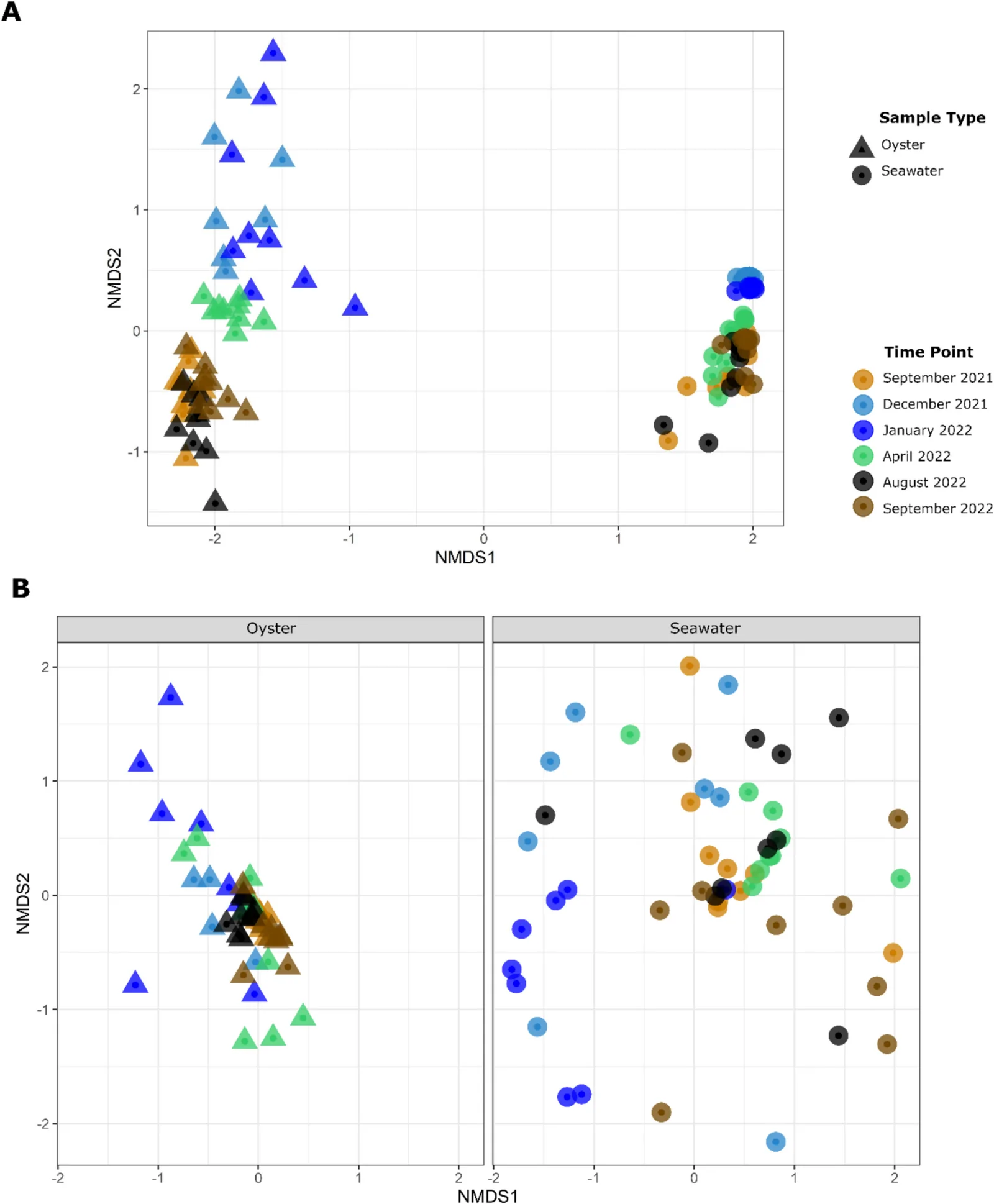
nMDS of bacterial (**A**) and *Vibrio* (**B**) communities in oysters and seawater at each time point. Wet season samples (November to April) are represented by light blue, dark blue and green, while dry season samples (May to October) are represented by orange, brown and black

The nMDS ordination showed distinct clustering of the oyster bacteriomes by season, with lower dispersion observed during the dry season (Fig. 3A). PERMANOVA results demonstrated significant differences between the bacteriome of the sample types (oysters versus seawater), as well as by time point and site (Table 2). Pairwise comparisons showed a significant difference in the oyster bacteriome during the dry season months *(p* = 0.001), and less differentiation in the wet season months of December and January (*p* = 0.066). While the PermDisp test indicated a significant difference in dispersion between the sample type, and by time point and site (*p* = 0.001), the significant PERMANOVA results reflected not only this variation in spread but also a clear separation in the community composition, as shown by the distinct clustering and different centroids in the nMDS ordination (Fig. 3A).

**Table 2:** Permutational multivariate analysis of variance (PERMANOVA) table for bacterial communities in oysters and seawater collected at 6 sample time points. ECV for square root estimates of the component of variation indicating the effect size as average percentage ASV dissimilarity due to that factor (residual ECV 56.3%)

Factor	Pseudo-F	df	*p-*value	ECV	PermDisp *p-*value
Sample Type (Oyster vs. Seawater)	87.5	1	0.001	48.4	0.001
Sample Time Point	6.6	5	0.001	21.2	0.966
Site	1.2	2	0.012	3.0	0.691
Sample Type by Time Point (Interaction)	5.4	5	0.001	6.6	0.001
Sample Type by Site (Interaction)	1.2	2	0.016	4.4	0.001

The vibriome in oysters was significantly different to the seawater vibriome (PERMANOVA *p* = 0.001) and also differed over time (PERMANOVA *p* = 0.001), but there was no significant difference between sites (PERMANOVA *p* = 0.115). Pairwise PERMANOVA testing demonstrated that *Vibrio* communities differed between oysters and seawater in all months (*p* < 0.010) except December 2021 (*p* = 0.24), which was likely due to the high dissimilarity in the seawater samples. PERMANOVA testing also showed that the oyster vibriome varied significantly between seasons (*p* = 0.001) and during the dry season months (*p* < 0.010), but not during the wet season months. The seawater vibriome displayed significant variation between seasons (*p* = 0.003) and all months except for some combinations of September, April and August 2022 (April 2022 – August 2022 *p* = 0.190; September 2021 – August 2022, *p* = 0.159). The oyster vibriome was similar across all dry season samples, with less dispersion according to location in the nMDS ordination (Fig. 3B).

### Taxa Contributing to Differences Between the Seawater and Oyster Bacteriome and Vibriome

No single bacterial family or *Vibrio* species was a key driver in the dissimilarity between seawater and oyster samples measured by SIMPER analysis. The top bacterial families contributed to a cumulative 5% dissimilarity were *Cyanobiaceae, Rhodobacteraceae* and *Flavobacteriaceae* in seawater, and *Mycoplasmataceae* and *Helicobacteraceae* in oysters.

SIMPER analysis showed that the top *Vibrio* species contributed to a cumulative 10% dissimilarity were *V. owensii*, *V. campbellii* and *V. harveyi*, all of which were more abundant in oysters. Differences between time points were mainly driven by the most abundant *Vibrio* spp*.* which were *V. owensii, V. harveyi, V. campbelli* or unidentified *Vibrio* spp. *Vibrio owensii* contributed to the top 10% cumulative dissimilarity between dry season September 2021 and wet season December 2021/January 2022 and were more abundant in the wet season. *Vibrio campbellii* and *V.* *harveyi* were both more abundant in September 2021.

### Seawater Chemistry, and Seasonal BRO Bacteriome and Vibriome Changes

**Fig. 4:**
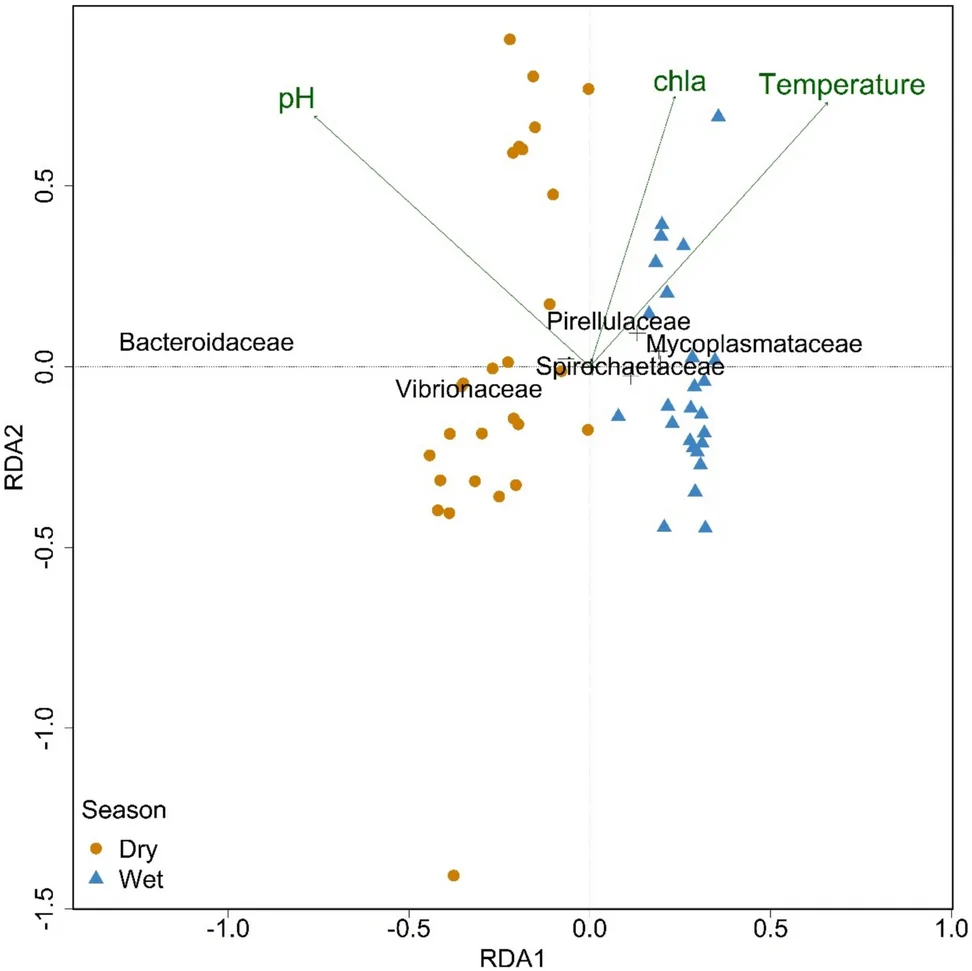
RDA correlation biplot of the bacterial community in oysters and environmental variables. Type II scaling was used with angles between vectors including families reflecting their linear correlation. The RDA model for 16S rRNA gene had an adjusted R^2^ of 52.2% and was largely driven by the 1st axis explaining 28.3% (*p* = 0.001) while the 2nd axis explained 0.7% (*p* > 0.1)

Changes in the oyster bacterial communities were significantly associated with the higher water temperatures (*p* = 0.001) and chlorophyll *a* levels (*p* = 0.016) observed in the wet season, as well as a more alkaline conditions (*p* = 0.001) during the dry season (Fig. 4). Turbidity and nutrients (TN and TP) had no significant influence on the bacterial community (*p* > 0.05). *Mycoplasmataceae* were associated with higher temperatures observed in the wet season while *Vibrionaceae* were associated with lower temperatures and increased pH in the dry season.

**Fig. 5:**
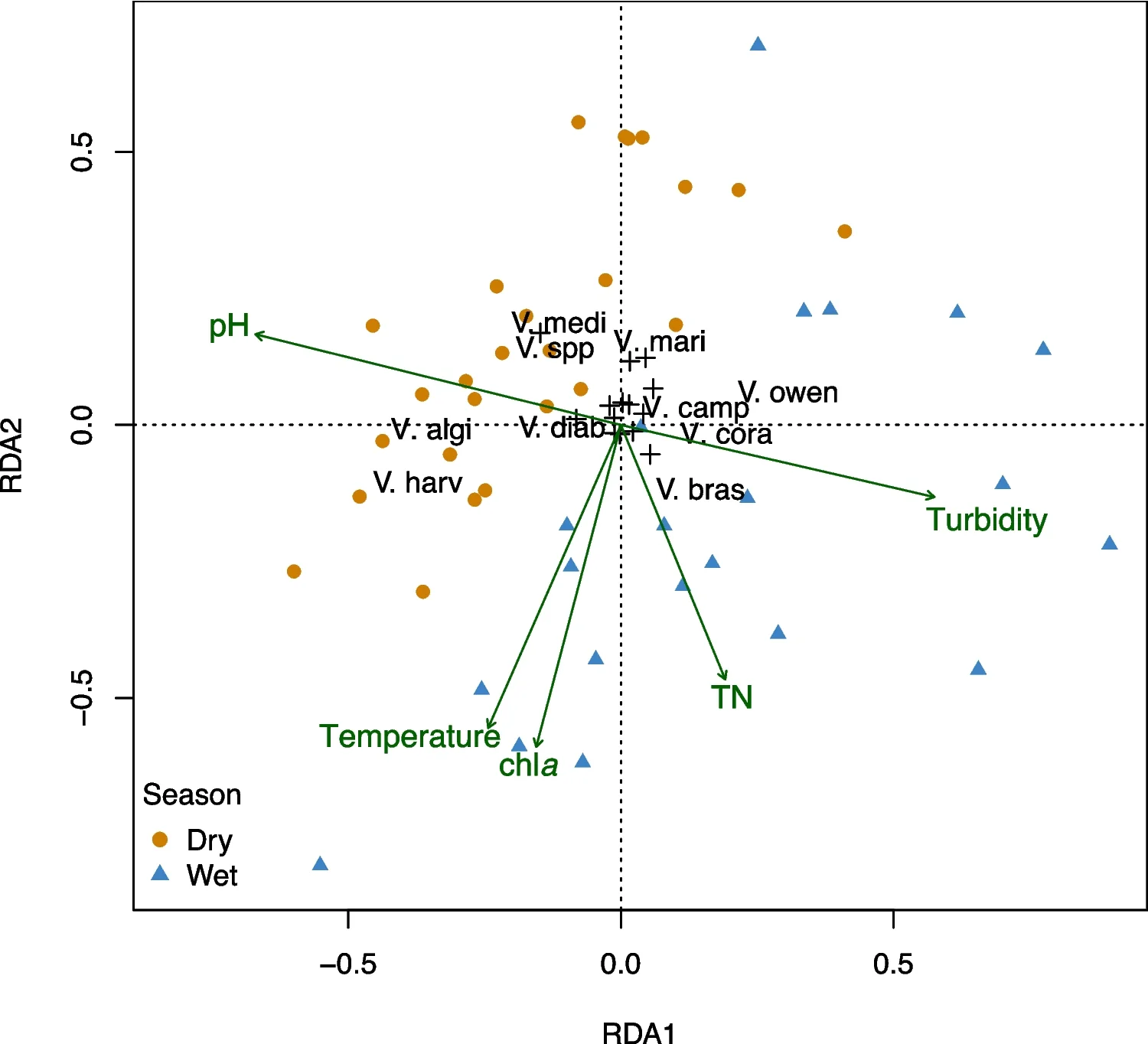
RDA correlation biplot of the *Vibrio* community in oysters, and environmental variables. Type II scaling was used with angles between vectors including species reflecting their linear correlation. *Vibrio* species abbreviations are *V. harveyi*, *V. coralliilyticus*, *V. brasiliensis*, *V.* *owensii*, *V. alginolyticus*, *V. campbellii*, *V. diabolicus*, *V. mediterranei* and *V. marisflavi*. The RDA explained 27.5% of the variability in the *Vibrio* community with the 1st axis explaining 15.7% (*p* = 0.001) and the 2nd axis 5.7% (*p* = 0.071)

Changes in the oyster *Vibrio* communities were associated with higher temperature, turbidity, chlorophyll *a*, TN and pH (all *p* < 0.050), but there was no strong association between particular *Vibrio* species and any of these environmental variables, as determined by redundancy analysis (Fig. 5). Relative abundance patterns of specific *Vibrio* species were compared across seasons using taxa bar plots (Fig. 2), which showed that *V. brasiliensis* was more abundant during the wet season, while *V. alginolyticus*, *V. harveyi*, and *V. mediterranei* were more abundant during the dry season.

### There Is a Core Bacteriome and Vibriome in Oyster Samples

Members of the core bacteriome and vibriome were defined as ASVs that occurred in at least 80% of all oyster samples. Overall, there were fifty-four core bacterial ASVs that occurred in 80% of all oyster samples which comprised nine bacterial families. The most abundant families within the core bacteriome were *Mycoplasmataceae, Cyanobiaceae, Vibrionaceae, Spirochaetaceae, Fusobacteriaceae* and *Helicobacteraceae.* At the genus level, the most abundant members of the core bacteriome were *Propionigenium*, *Mycoplasma, Tenacibaculum* and *Vibrio.* Based on the *hsp60* data, there were three *Vibrio* ASVs that occurred in 80% of all oyster samples, all three ASVs were classified as *V. owensii*. At species level, *V. harveyi* was also present in 82% of oyster samples, represented by 39 ASVs.

### Potential Bacterial and Vibrio Pathogens in Seawater and Oysters Change Seasonally

**Fig. 6:**
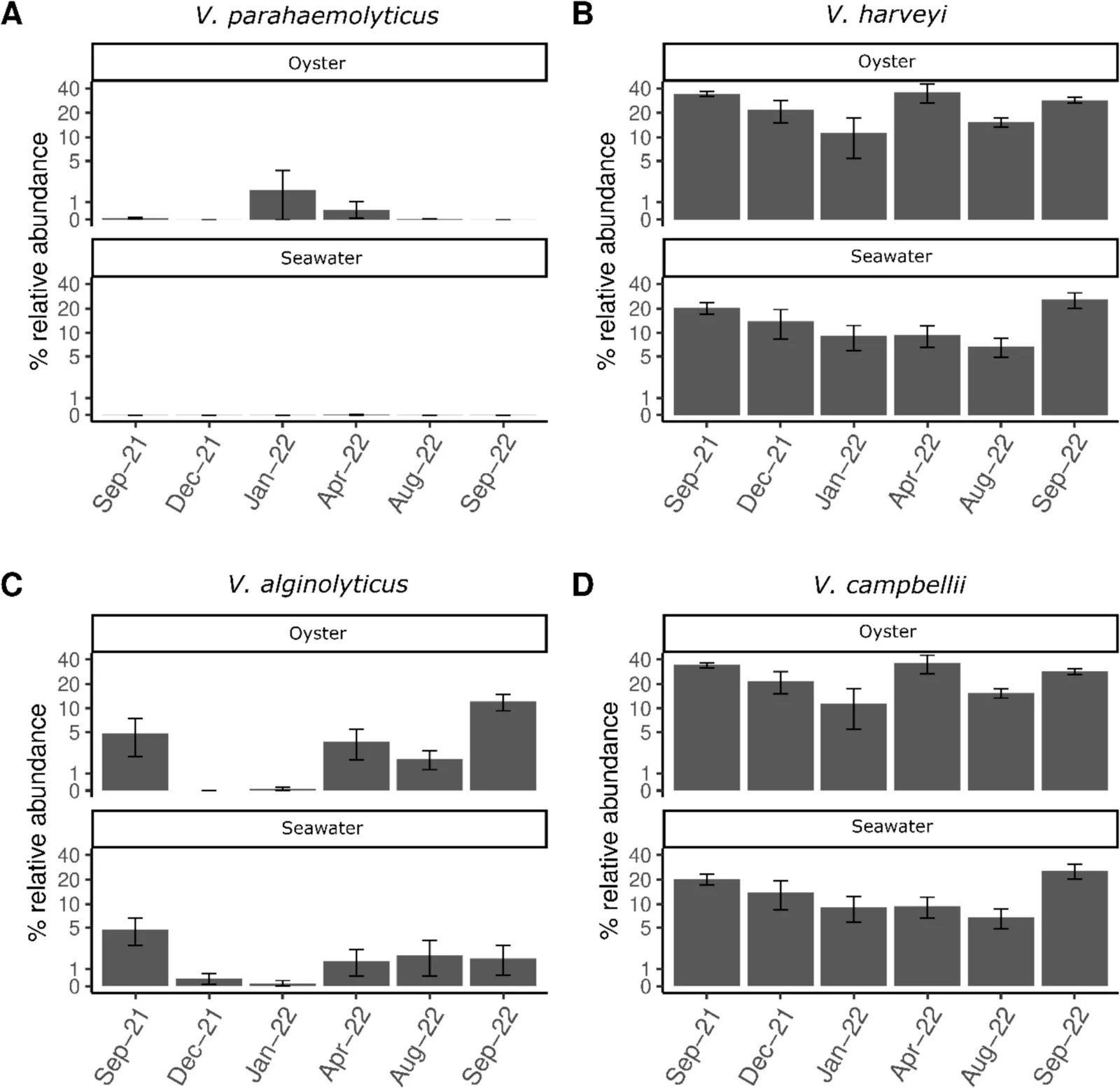
Bar plots of the average relative abundance of known potential human and oyster *Vibrio* pathogens detected in oysters and seawater at different sample times. The error bars mark 1 × standard error. The y-axis is displayed on a log10-scaled axis to improve resolution of lower-abundance taxa while maintaining values in percent

The most abundant potential human pathogens in oysters and seawater included members of the *Vibrio* and *Bacteroides. Vibrio* spp. were detected in 99% of all samples with a mean relative abundance ranging from 0.3 to 23.8%. The relative abundance of *Vibrio* spp. in oysters was significantly higher than in seawater (Mann–Whitney U test *p* < 0.001). *Bacteroides* spp*.* were detected in 61% of all samples with a mean relative abundance ranging from 0.1 to 48.3% but were most prevalent in oysters. Other potential oyster and human pathogens such as *Roseovarius* and *Pseudoalteromonas* were detected in low abundance (Supplementary Table 2). Within the *Vibrio* genus, potential human pathogens were detected in oysters including *V. parahaemolyticus* (0.18%), *V. alginolyticus* (1.24%) and *V. fluvialis* (0.02%) (Supplementary Table 1). *Vibrio parahaemolyticus* abundance was highest in some wet season oysters (Fig. 6A), however the strain was not identified and therefore the pathogenicity of *V. parahaemolyticus* in BROs is not known. *Vibrio alginolyticus,* in contrast to *V. parahaemolyticus,* had higher relative abundance during the dry season (Fig. 6C).

The potential oyster pathogen *V. harveyi* (Fig. 6B) was present in both oysters and seawater, and was a member of the BRO core vibriome. The potential oyster pathogen *V. campbellii* (Fig. 6D) was also present in both oysters and seawater and, although not a member of the core vibriome, was detected in 72% of oyster samples.

## Discussion

This is the first baseline examination of the microbial communities associated with BROs, providing insight into the temporal patterns in the BRO microbiome which has the potential to inform future pathogen monitoring strategies for farm management, to ensure shellfish meet food safety quality assurance standards. We have shown that the BRO bacteriome and vibriome changed significantly over time, except in the wet season months of December 2021 and January 2022. Such changes in the oyster microbiome are consistent with patterns reported in other tropical oyster species *Crassostrea gasar* [31] and temperate oyster species *C. gigas* and *C. virginica* [7, 8, 30, 32]. In this study, there were significant differences in the bacteriome, but not the vibriome, between sites within the BRO farm. This is consistent with findings by Lokmer et al. [8], who noted that microenvironmental variation can drive bacterial differences within a single location.

Even though the oyster microbial communities changed over time and by site, a core BRO bacteriome and vibriome was still apparent that comprised of ASVs spanning nine bacterial families, and two *Vibrio* species. Members of both the *Mycoplasmataceae* (*Mycoplasma* genus) and *Spirochaetaceae* were identified as core members of the BRO bacteriome, which is consistent with reports in other oyster species, including tropical rock oysters from multiple sites across northern Australia [24], and the temperate oyster species *C. gigas* [6, 7, 30], *C. virginica* [32] and *Ostrea lurida* [9]. This suggests the presence of a conserved core microbiome across both tropical and temperate oysters. In addition, the *Mycoplasma* have previously been identified as members of the core microbiome for oysters spanning all life stages [33]. Increases in the occurrence of potential oyster pathogens have previously been linked to a reduction in the relative abundance of *Mycoplasma,* pointing to their potential utility as an indicator of oyster health [30, 34]. In this study, increases in *Mycoplasmataceae* were associated with increases in turbidity, temperature and nutrients during the wet season. A reduction in the relative abundance of *Mycoplasmataceae,* which notably coincided with an increase in the relative abundance of potentially pathogenic families, including *Vibrionaceae* and *Bacteroidaceae,* was also observed in the BRO microbiome during the dry season.

Interestingly, the core taxa described in this study included potential human faecal indicators *Bacteroidaceae* and *Helicobacteraceae* supporting previous work [24]*.* In the present study, *Bacteroides* was highest during the dry season, when oyster condition is optimal for human consumption. This seasonal pattern is consistent with tropical oyster species *C. gasar*, where *Bacteriodes* was also associated with the dry season [31]. In contrast, other faecal indicator bacteria such as *Helicobacteraceae* and *Escherichia-Shigella* were highest in the wet season, after periods of increased rainfall. Other potential faecal indictor bacteria such as *Enterobacteria* and *Aeromonas* were not detected, which suggests the bacteria in the present study came from a source other than human. There is no certainty that these core families do contain pathogenic species so this needs to be resolved in subsequent studies, particularly in relation to their presence and relationship with BROs.

Our results demonstrate that members of the *Vibrionaceae* are core members of the BRO microbiome, with low variability in the oyster vibriome during the dry season which reflect patterns in the seawater vibriome throughout the year. This finding aligns with previous studies in both tropical and temperate oyster species, where *Vibrio* spp. have also been identified as persistent members of the oyster microbiome [9, 24]. In our study, *Vibrio* spp. were present in 99% of oyster and seawater samples, with two core species, *V. harveyi* and *V. owensii,* identified within 80% or more of samples*.* Several *V. harveyi* ASVs were identified, highlighting the genetic diversity within this species, a topic reviewed by Montánchez and Kaberdin [35]*.* This is notable because *V. harveyi* has regularly been linked to mass mortalities in other oyster species [11, 12, 36] and, due to similarities in the BRO microbiome to other oyster species, *V. harveyi* could be a threat to BRO health. *Vibrio owensii* and *V. campbellii* are also members of the Harveyi clade and, similarly, are emerging marine pathogens [37, 38], both of which have been associated with Pacific Oyster summer mortalities [39]. However, in some studies, *V. owensii* has been implicated in oyster disease [40], while in others it has been shown to be potentially beneficial [41]. The functional role and pathogenicity of these identified *Vibrio* spp*.* warrants further investigation in subsequent studies.

In our study, the potential human pathogen *V. parahaemolyticus* occurred in high relative abundance in some oyster samples during the wet season, when temperature and chlorophyll *a* levels were higher. Increased *V. parahaemolyticus* abundance was also associated with increases in temperature, turbidity, salinity and chlorophyll *a* in other systems [17, 42–44]*.* Cyanobacteria are also reportedly associated with high levels of *V. parahaemolyticus* [30] and indeed, increases in *Cyanobiacae* occurred in January 2022 when *V. parahaemolyticus* abundance was highest. Therefore, easily measurable environmental parameters, such as temperature and chlorophyll *a*, could be employed as an early warning test for this potential human pathogen. This is particularly relevant given that current Australian Shellfish Quality Assurance Program (ASQAP) guidelines [44] primarily focus on monitoring enteric human pathogens such as *Escherichia coli*, norovirus, and hepatitis A [45], and do not adequately capture the dynamics of *Vibrio* spp. and associated oyster and human health risks. In remote locations, tools that can be simply and affordably applied to help inform oyster growers of ‘high-risk’ food safety periods will be highly beneficial. In northern Australia, remoteness and high temperatures highlight the need for additional studies to assess the effect of storage temperature on *V. parahaemolyticus* abundances in Blacklip Rock Oysters.

Notably the temporal dynamics of the BRO bacteriome and vibriome were significantly different to the surrounding seawater, so routine surveillance of seawater will not necessarily cover all pathogenic targets like *V. parahaemolyticus*. However, while the relative abundance of some other important human and animal health target species such as *V. harveyi* and *V. alginolyticus* differed with season, they had a similar relative abundance in oysters and seawater. These targets therefore could potentially be predicted according to changes in seawater conditions. Data from the current study demonstrates that periods of increased rainfall, turbidity and total nitrogen during the wet season months indicate potentially higher risk periods when *V. parahaemolyticus* increases in abundance.

## Conclusion

In this study we report temporal patterns in the bacterial communities associated with BROs in northern Australia and have identified potential risks to human and animal health. Our results provide important information to support decision-making about food safety by identifying locally relevant oyster and human pathogen targets such as *Vibrio harveyi*, *V. campbellii*, *V. alginolyticus* and *V. parahaemolyticus,* for routine surveillance. Furthermore, increases in rainfall, turbidity and total nitrogen during the wet season appear to correspond to ‘high risk’ periods when *V. parahaemolyticus* is most abundant. This work contributes important new insights into the microbiology of tropical oysters. Future work should build on these findings to develop targeted pathogen monitoring to form the basis of an early warning surveillance method for remote communities across northern Australia and the seasonal tropics.

## Supplementary Information

Below is the link to the electronic supplementary material.

Supplementary file1 (DOCX 42.0 KB)

## Data Availability

Raw sequence files are accessible through NCBI Sequence Read Archive (PRJNA1264689).
