# Supplementary material for: Dynamics of Bacterial and Vibrio Communities in Blacklip Rock Oysters in the Seasonal Tropics
Source: Microb Ecol. 2025 Nov 18;88(1):125. doi: 10.1007/s00248-025-02599-w (PMC12628417; doi:10.1007/s00248-025-02599-w)
Supplement: Supplementary file 1 — Supplementary file1 (DOCX 42.0 KB) [file 248_2025_2599_MOESM1_ESM.docx]

**Supplementary Information**

**Figure S1.** Monthly rainfall for Warruwi Airport, South Goulburn Island, 2021-2022 (Bureau of Meteorology, accessed 18 July 2023). Red diamonds represent the sampling dates.


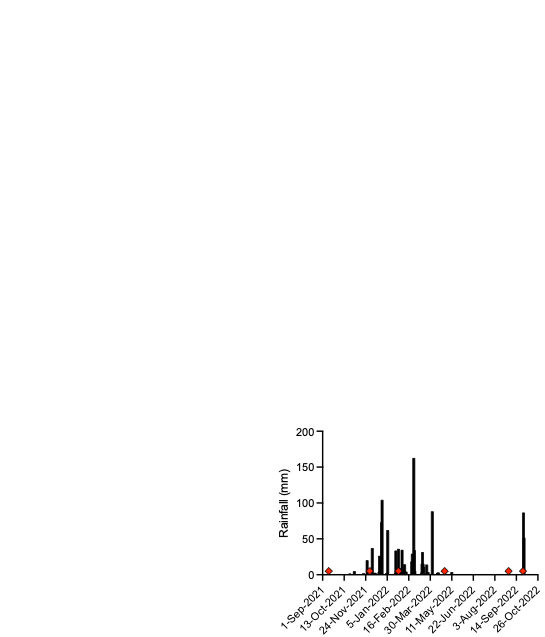


**Table S1.** *Vibrio* species identified in BROs and seawater, and their relative abundance**.**

| **Species** | **Relative abundance** |
| --- | --- |
| *V. owensii* | 26.27 |
| *V. harveyi* | 19.15 |
| *V. spp* | 15.84 |
| *V. brasiliensis* | 12.10 |
| *V. campbellii* | 5.83 |
| *V. coralliilyticus* | 5.54 |
| *V. rotiferianus* | 3.28 |
| *V. alginolyticus* | 2.95 |
| *V. sinaloensis* | 2.06 |
| *V. mediterranei* | 1.01 |
| *V. fortis* | 0.82 |
| *V. tubiashii* | 0.66 |
| *V. diabolicus* | 0.42 |
| *V. mexicanus* | 0.42 |
| *V. panuliri* | 0.34 |
| *V. maritimus* | 0.30 |
| *V. ponticus* | 0.28 |
| *V. variabilis* | 0.25 |
| *V. ishigakensis* | 0.24 |
| *V. parahaemolyticus* | 0.21 |
| *V. neptunius* | 0.18 |
| *V. marisflavi* | 0.14 |
| *V. thalassae* | 0.14 |
| *V. aerogenes* | 0.11 |
| *V. nereis* | 0.11 |
| *V. hepatarius* | 0.07 |
| *V. natriegens* | 0.06 |
| *V. alfacsensis* | 0.05 |
| *V. sonorensis* | 0.04 |
| *V. nigripulchritudo* | 0.03 |
| *V. xuii* | 0.03 |
| *V. fluvialis* | 0.02 |
| *V. cidicii* | 0.01 |
| *V. splendidus* | 0.01 |
| *V. orientalis* | 0.01 |

**Table S2.** List of potentially pathogenic bacterial genera and mean relative abundance in seawater and oyster samples.

| **Genus** | **Potential Human or Animal Pathogen?** | **Mean relative abundance range (%)** | **No. of Samples (%)** | **Reference** |
| --- | --- | --- | --- | --- |
| *Amphritea* | Animal | 0.1-1.9 | 49 | [46] |
| *Bacteroides* | Human | 0.1-48.3 | 61 | [47] |
| *Escherichia-Shigella* | Human | 0.1-0.5 | 34 | [49] |
| *Marinobacterium* | Animal | 0.1 | 42 | [46], [48] |
| *Marinomonas* | Animal | 0.1-2.1 | 57 | [46], [48] |
| *Photobacterium* | Animal | 0.1-2.1 | 86 | [50] |
| *Pseudoalteromonas* | Animal | 0.1-7.4 | 81 | [51], [52] |
| *Roseovarius* | Animal | 0.1 | 27 | [53] |
| *Shewanella* | Human & Animal | 0.1 | 53 | [50], [52] |
| *Vibrio* | Human & Animal | 0.3-23.8 | 99 | [46], [49], [52] |
